# Elucidation of the bonding of a near infrared dye to hollow gold nanospheres – a chalcogen tripod†
†Electronic supplementary information (ESI) available. See DOI: 10.1039/c6sc00068a


**DOI:** 10.1039/c6sc00068a

**Published:** 2016-04-21

**Authors:** H. Kearns, S. Sengupta, I. Ramos Sasselli, L. Bromley III, K. Faulds, T. Tuttle, M. A. Bedics, M. R. Detty, L. Velarde, D. Graham, W. E. Smith

**Affiliations:** a Department of Pure and Applied Chemistry , Technology and Innovation Centre , University of Strathclyde , 99 George Street , Glasgow G1 1RD , UK . Email: duncan.graham@strath.ac.uk; b Department of Chemistry , University at Buffalo , Buffalo , NY 14260 , USA . Email: lvelarde@buffalo.edu

## Abstract

Infrared surface enhanced Raman scattering (SERS) is an attractive technique for the *in situ* detection of nanoprobes in biological samples due to the greater depth of penetration and reduced interference compared to SERS in the visible region. A key challenge is to understand the surface layer formed in suspension when a specific label is added to the SERS substrate in aqueous suspension. SERS taken at different wavelengths, theoretical calculations, and surface-selective sum frequency generation vibrational spectroscopy (SFG-VS) were used to define the surface orientation and manner of attachment of a new class of infrared SERS labels with a thiopyrylium core and four pendant 2-selenophenyl rings. Hollow gold nanospheres (HGNs) were used as the enhancing substrate and two distinct types of SERS spectra were obtained. With excitation close to resonance with both the near infrared electronic transition in the label (max 826 nm) and the plasmon resonance maximum (690 nm), surface enhanced resonance Raman scattering (SERRS) was obtained. SERRS indicates that the major axis of the core is near to perpendicular to the surface plane and SFG-VS obtained from a dried gold film gave a similar orientation with the major axis at an angle 64–85° from the surface plane. Longer excitation wavelengths give SERS with little or no molecular resonance contribution and new vibrations appeared with significant displacements between the thiopyrylium core and the pendant selenophene rings. Analysis using calculated spectra with one or two rings rotated indicates that two rings on one end are rotated towards the metal surface to give an arrangement of two selenium and one sulphur atoms directly facing the gold structure. The spectra, together with a space filled model, indicate that the molecule is strongly adsorbed to the surface through the selenium and sulphur atoms in an arrangement which will facilitate layer formation.

## Abstract 2

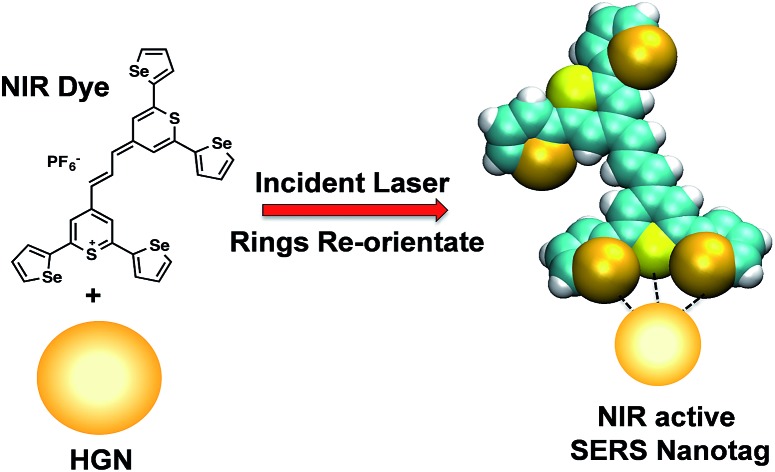
Determining how Raman labels orientate on the surface of HGNs to aid in future advancements of designing NIR nanosensors.

## Introduction

The near infrared (NIR) region of the electromagnetic spectrum is particularly suitable for the use of SERS to detect metallic nanoprobes in biological samples, such as tissue, due to the greater depth of sample penetration compared to the visible region.^1^–^3^ To exploit this, *in situ* identification of each probe in a mixture is highly desirable and can be related to different biomolecules or functions. Raman scattering offers good molecular specificity but the 4^th^ power of the frequency dependence means that the signals tend to be weak in the infrared (IR). However, the intensity of the scattering from surface enhanced Raman scattering (SERS) is dependent on coupling with the plasmon from the enhancing surface and can be intense well into the infrared.^4^–^6^ In addition, SERS enhancement can produce signals several orders of magnitude more intense than Raman scattering with pyridine, for example, giving an enhancement of 10^6^.^7^–^9^ When a dye is used as the label, even greater enhancements may be obtained. With rhodamine dyes, enhancement factors between 10^13^ and 10^15^ have been previously reported.^10^–^12^ This form of SERS is often labelled as surface enhanced resonance Raman scattering (SERRS) and in this paper the spectra obtained in and out of molecular resonance are quite different indicating different selection rules for the two forms.

Recently a SERS probe consisting of hollow gold nanospheres (HGNs) labelled with a new class of infrared dyes with pendant selenophene rings was found to be effective with 1280 nm excitation.^13^ The importance of this observation is that the selenophene rings can give strong surface attachment and modification of the dye substituents alters the SERS spectrum significantly, making it possible to create a range of probes to label several molecular targets and detect them *in situ* at this IR wavelength.

One key requirement to inform the design of dye labels is to understand the geometry and bonding of the label on the SERS substrate in suspension in aqueous media. This study aims to understand the nature of the attachment of one of these new infrared dyes to an HGN surface. SERS has the potential to provide significant information and this paper reports a theoretical and practical study of resonance and SERS/SERRS data obtained with four excitation wavelengths between 633 nm and 1280 nm. However, determining with certainty the nature of attachment of a label *in situ* is difficult and consequently a second technique, surface-selective sum frequency generation vibrational spectroscopy (SFG-VS), a well-established surface analysis tool for determining the molecular orientation distribution of organic ligands adsorbed on surfaces and nanoparticles, was applied for comparison and to determine more precisely the orientation of the dye at the interface. This is the first time this level of molecular characterisation has been obtained for this type of surface ligand and provides a unique insight into the surface chemistry with gold that will be significant in shaping future spectroscopic and chemistry studies.

## Experimental

### Synthesis of hollow gold nanotags

**Fig. 1:**
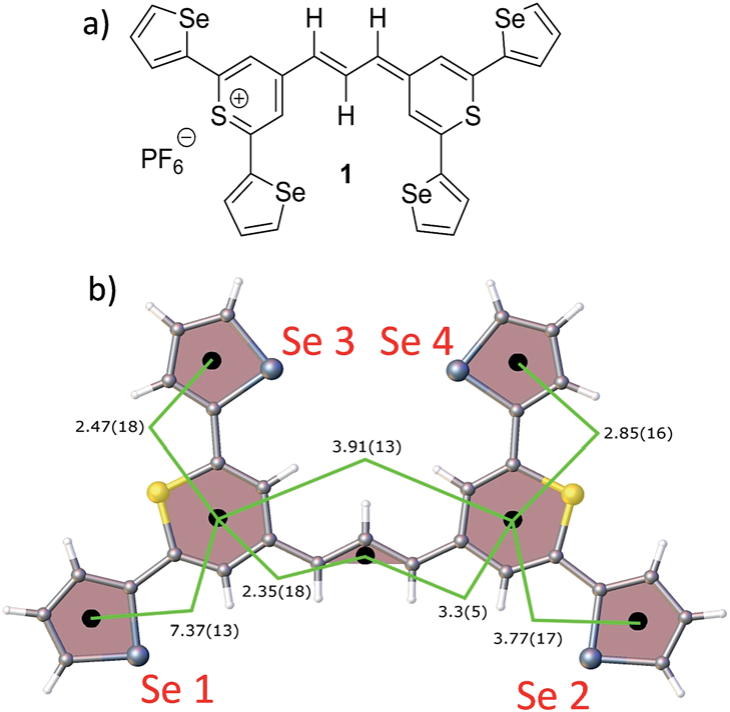
(a) Molecular structure of thiopyrylium dye **1**, (b) structure of dye **1** with ring numbers and showing the torsion angles. The two thiopyryium sulphur atoms are colour-coded yellow.

The hollow gold nanotags were synthesised and characterised as reported in a previous publication by Bedics *et al.*^13^ They have a localised surface plasmon resonance (LSPR) at 690 nm and a final concentration of 3 nM. The thiopyrylium Raman reporter (dye **1**, Fig. 1a) was prepared as described in reference.^13^ Dye **1** (8.5 mg, 9.5 × 10^–6^ mol) was dissolved in 2.5 mL of dimethylformamide (DMF) to yield a concentration of 3.8 mM. Subsequent dilutions to 10 μM were prepared in 1 : 1 ratios of DMF to deionised water.

### Raman and SERS characterisation

Raman spectra were acquired from ∼5 mg of solid sample spread on a microscope slide. The Raman measurements were performed using two Renishaw InVia Raman microscope systems, equipped with 50× objectives. Three excitation sources were employed; an Ar^+^ (argon ion) laser with 514.5 nm excitation giving 0.825 mW, a He–Ne (helium–neon) laser with 632.8 nm excitation giving 0.525 mW and a diode laser with 785 nm excitation and 0.345 mW. Acquisition times were 10 s at 514.5 nm and 25 s at 632.8 nm and 785 nm.

SERS samples were prepared by adding an HGN suspension (135 μL; 3 nM) to solutions of the Raman reporter (20 μL; 10 μM) and potassium chloride (150 μL; 30 mM). For the 1280 nm SERS analysis the volume of each component was doubled.

633 nm and 785 nm SERS measurements were taken using the same instruments, laser power and objectives as described for the Raman analysis and with 10 s acquisition times. Samples were analysed through transparent bottom microtitre plates with 300 μL of the nanotag solution placed in each well. SERS measurements further into the infrared were obtained from suspensions in glass cuvettes using a Real Time Analyser FT-Raman spectrometer with a 1064 nm Nd:YAG laser and a Snowy Range portable Raman spectrometer with a 1280 nm diode laser. The laser powers were 420 mW and 100 mW and acquisition times were 5 and 3 s, respectively. All spectra have been background corrected.

The Raman measurements were normalised to a silicon standard with respect to the peak at (520 ± 2) cm^–1^. For SERS, a cyclohexane spectrum was obtained for each excitation wavelength using the same laser power, accumulation time and objective as was used for the SERS spectra. Peak wavenumber positions and relative intensities of the peaks within each spectrum were similar in all cases indicating that the frequency response of each instrument was well calibrated. The cyclohexane response was then used to normalise resonance and SERS data for each wavelength using the 801 cm^–1^ cyclohexane peak and the strongest SERS peak at about 1590 cm^–1^. The ratio was not corrected for the fourth power dependence of the normal Raman scattering from cyclohexane. This method leaves an error estimated at about 2 cm^–1^ between spectra.

Throughout, the Raman peaks which are discussed in detail have been labelled accordingly on the relevant Raman spectra; however, Table S1 in the ESI† provides detailed assignments of all the Raman and SERS peaks.

### DFT calculations

All calculations were performed in Gaussian 09^14^ with the Def2-TZVP^15^,^16^ basis set. The DFT functional B3LYP^17^–^20^ was used for the structure optimisation, Raman activity calculations and for torsion profile calculations of the rotation of ring 1 and ring 3 (Fig. S1 in ESI†). Theoretical electronic spectra were calculated using TD-DFT^21^–^24^ with the cam-B3LYP.^25^ The crystal structure geometry was used for the calculation of the electronic spectrum. Raman spectra were calculated for the crystal structure geometry and with ring 1, ring 3 and rings 1 and 3 rotated. Conformations where obtained by rotating the rings ∼180 degrees (see Fig. 1b for ring numbers). The calculated Raman activities (*S*_*i*_) were converted to Raman intensities (*I*_*i*_) using the equation:^26^–^30^ 
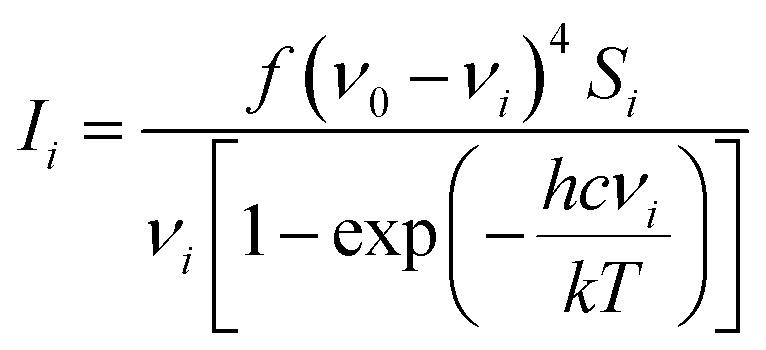
where *ν*_0_ is the exciting wavenumber, *ν*_*i*_ is the wavenumber of the mode *i*, *h* is Planck's constant, *c* is the speed of light, *k* is Boltzmann's constant, *T* is the temperature and *f* is a proportionality constant. The calculated Raman frequencies were corrected to fit the experimental mode at 1588 cm^–1^ Raman prediction (correction factor: 0.968). Zoomed calculated spectra are included in the ESI for clarity (Fig. S3†).

### Sum frequency generation vibrational spectroscopy

To prepare samples for surface-selective sum frequency generation vibrational spectroscopy (SFG-VS), a solution of 600 μM of dye **1** in acetonitrile was spin cast at 4000 rpm onto a clean polycrystalline gold film (100 nm thick). The gold substrates were cleaned by sonication (10 min in acetone, detergent, and 18.2 MΩ water each), dried by a flow of dry nitrogen and subsequently heated at 110 °C for 1 h. A home-built broadband SFG spectrometer^31^ was used for the measurements. In brief, a total of 3 mJ of the output of a regenerative amplified Ti:sapphire laser system (Legend HE+, Coherent) producing ∼35 fs pulses at 1 kHz centered at 800 nm is used to generate mid-IR pulses in an optical parametric amplifier (OPA) equipped with a difference frequency generation unit (Topas PRIME and NDFG, Light Conversion). A total of 1.7 mJ of the amplifier output is used to generate ∼5 ps pulses at 400 nm (SHBC, Light Conversion), which pump an OPA to generate narrowband pulses (∼7 cm^–1^) at 694.2 nm. The mid-IR and visible (VIS) pulses are overlapped spatially and temporally at the sample surface with angles of incidence of 70° and 45° with respect to the surface normal and average powers of 4.5 mW and 1 mW, respectively. After interaction with the sample, the VIS and mid-IR beams are filtered out by a 650 nm short-pass filter, and the optical signal at the sum frequency is collected by an imaging spectrograph (Princeton). The spectra presented here are the result of an average of three 5 min acquisitions. Each spectrum is background subtracted and normalised by acquisition time and with respect to the broad non-resonant signal of a bare gold film. The 1601.35 cm^–1^ line of a polystyrene film was used for calibration. The mid-IR path and the sample are continuously purged with dry air to alleviate atmospheric absorption of the mid-IR beam.

## Results and discussion

### Structure and electronic structure of dye **1**

The crystal structure of the label has the four selenophenyl rings oriented as shown in Fig. 1b so that the molecule is essentially flat with some out of plane twisting of the rings. The angles shown by the green lines indicate the degree of this twist. One ring shows a larger out of plane rotation of 7.37 degrees. There are 4 molecules per unit cell and there is 2.5 to 7% disorder in the structure mainly due to the orientation of the rings and in particular the rings containing Se2 and Se3. Calculation of the barriers to rotation of rings Se1 and Se3 shows that they are very small at about 4 kcal (Fig. S1†) and consequently in solution or attached to the HGN surface the orientation of the rings will depend on other forces such as those created by bonding and packing on the surface.

The electronic spectrum (Fig. S2a in ESI†) has a strong band with a peak at 826 nm which is due to a π to π* transition between orbitals with the electron density mainly along the major axis of the thiopyrylium core (Fig. S2c†). It should be noted that this calculation was for a gas phase molecule and had a limited basis set. The wavelength dependence of the plasmon is also shown in Fig. S2b.† It has a maximum at 690 nm and a tail into the infrared with no indication of Fano resonances up to the limit available of 1100 nm. The effect of adding the dye to the HGN and the effect of aggregation carried out to increase SERS signals is a reduction in intensity but with little change in the wavelength of the peak maximum.

### Resonance Raman scattering

**Fig. 2:**
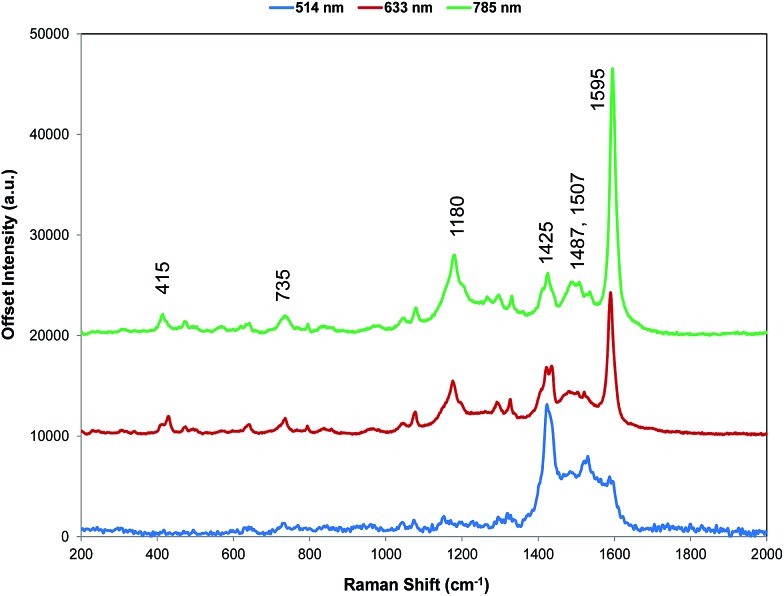
Resonance Raman spectra for dye **1** at three excitation wavelengths (514, 633, and 785 nm). The 514 nm spectrum is multiplied by a factor of 10 for clarity. The 785 nm Raman bands which are discussed throughout have been labelled accordingly but for a detailed interpretation of all peaks, see Table S1, ESI.†

For comparison with SERS, Raman scattering from dye **1** was recorded with 514, 633, and 785 nm excitation. The spectrometers used to obtain 1064 and 1280 nm spectra were not set up for the measurement of small quantities of solid and we were unable to obtain any Raman spectra. The spectra that were obtained are all resonant or preresonant (Fig. 2). There is clear evidence of selective enhancement, the intensities do not depend on the fourth power of the frequency, and spectra taken up to 3500 cm^–1^ showed no evidence of C–H stretching modes, which would have been quite intense in normal Raman scattering. Using cyclohexane standards as the reference, the band at 1595 cm^–1^ with 785 nm excitation is about twice as intense as with 633 nm excitation as would be expected for resonance or pre-resonance with the electronic transition with a visible absorption maximum at 826 nm. The spectrum obtained at 514 nm is weaker but it is still resonant and gives a different enhancement pattern due to an interaction with the transition at about 500 nm.

**Fig. 3:**
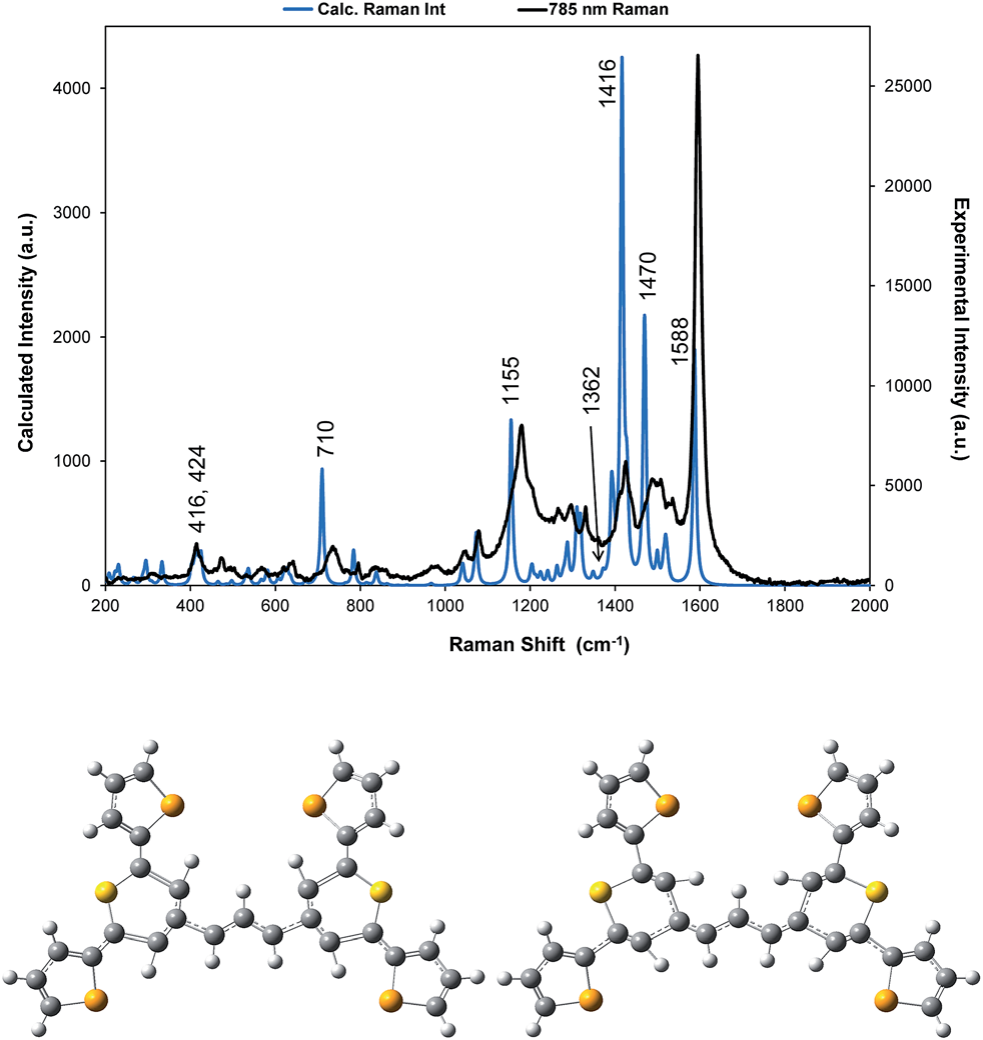
For dye **1**, comparison of the 785 nm resonance Raman spectrum with the corrected theoretical spectrum and displacement diagrams for the 1588 cm^–1^ vibration. The predicted Raman bands which are discussed throughout are labelled accordingly but for all band positions and assignments see Table S1.†

**Fig. 4:**
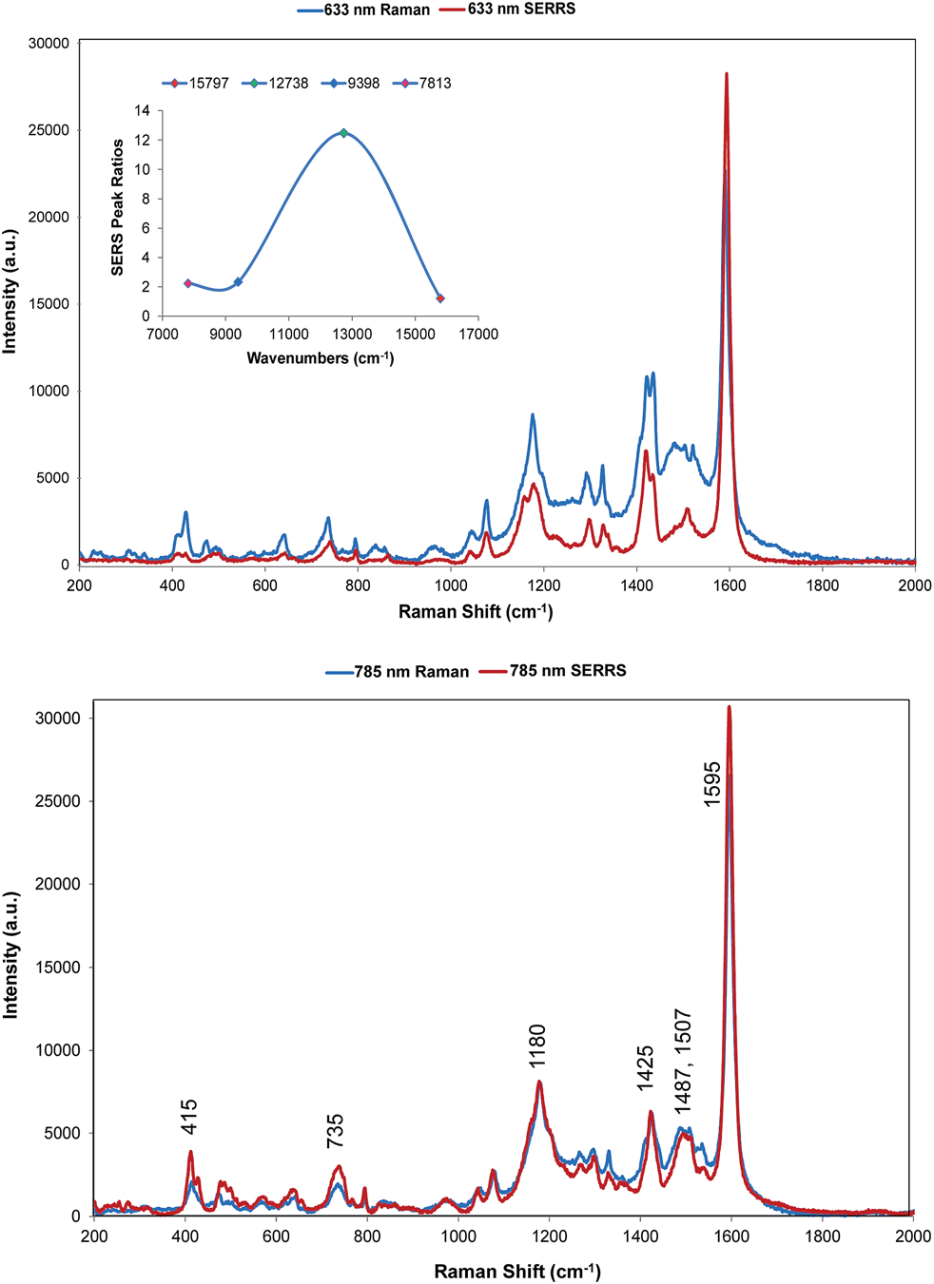
SERRS of dye **1** obtained with 633 nm (top) and 785 nm (bottom) excitation compared to resonance Raman spectra. Resonance spectral intensities have been set to show the difference with SERRS. Actual intensities are indicated in Fig. 2. The 785 nm Raman bands which are discussed throughout have been labelled accordingly. The inset shows the relative intensities of the 1588 cm^–1^ band in the SERRS spectra compared to cyclohexane standards. The intensities are plotted against excitation wavelengths converted to wavenumber to obtain a linear energy scale (1280 nm = 7813 cm^–1^ and 633 nm = 15 797 cm^–1^).

SERS from gold surfaces using 514 nm excitation is weak, as would be expected for a gold substrate,^32^ and hence the 514 nm resonance spectrum is only considered as it affects the 633 and 785 nm spectra. The spectrum obtained with 785 nm excitation is the most intense since it is closest in energy to the 826 nm absorption band (see Fig. 4 and S2†). The 633 nm excitation frequency is 3058 cm^–1^ higher in energy and gives preresonant enhancement with some changes occurring in the spectrum The band at 1425 cm^–1^ is relatively more enhanced in the 633 nm spectrum than in the 785 nm spectra and is a doublet. A band close to this frequency is strongly enhanced in the 514 nm spectrum which obtains its enhancement from a higher energy transition and there are two vibrations and a shoulder which are predicted theoretically to have significant Raman intensity in this region (Fig. 3). Thus, it is likely that the doublet in the 633 nm spectrum is due to pre-resonance enhancement of two closely spaced bands, which are enhanced by different transitions.

A correction factor was applied to the frequency of the calculated Raman spectrum to fit the highest energy band calculated as intense to the highest energy experimental SERS band at 1588 cm^–1^. The calculation was carried out for a gas phase molecule and is used here both for comparison with resonance Raman data obtained from solid state samples and SERS spectra taken from the surface of the HGNs in suspension. Different types of vibration observed in the experimental spectra will be affected differently both by shortcomings in the basis set and by changes in environment so some difference between the calculated and experimental shifts is expected. However the differences are sufficiently small to enable assignment of the main bands and enable comparisons between spectra. A comparison of the spectrum taken with 785 nm excitation with the predicted spectrum is given in Fig. 3 and an assignment of the bands observed in the resonance and SERS spectra are given in Table S1 in the ESI.†

The displacements in the most intense band at 1595 cm^–1^ (1588 cm^–1^ in the theoretical spectrum) are shown in Fig. 3 by depicting the extreme positions of the vibration. The major displacements are in the S-containing (thiopyrylium) rings extending into the bridge between them. Neglecting symmetry, resonance enhancement is strongest where there is significant electron density and the vibrational displacements take the ground electronic state geometry into the excited state geometry so that there is an electronic overlap at all positions of the displacement.^32^,^33^ This is clearly the case for this vibration (see Fig. S2† for the electron distribution in the ground and excited states). The 1425 cm^–1^ and 1180 cm^–1^ modes also have bridge mode and S-ring displacements along the major axis with additional displacements extending onto selenophene ring modes 1 and 2 (1425 cm^–1^) and 3 and 4 (1180 cm^–1^). In the experimental spectra, there is evidence of broadening of the bands particularly with the 1180 cm^–1^ mode probably due to the different molecular environments in the crystal. The gas phase calculation pairs the selenophene rings together to a large extent but the environment in the crystal is not exactly the same for each pair and this disorder may account for the additional structure in the experimental spectra. Later the effect of ring orientation is examined theoretically to explain the SERS spectra and these calculations confirm the above conclusion. The 1470 cm^–1^ mode, which is weak in the experimental spectrum, has some bridge displacement but it is not along the major axis and does not efficiently take the ground state into the excited state. Lower frequency spectra give a reasonable fit with the predicted spectrum (Fig. S3†). The band at 735 cm^–1^ is mainly a S-containing ring breathing mode and is displaced in the corrected calculation. Without the correction the fit is much closer, illustrating the different effect of the environment on different types of vibration. The broad band at 415 cm^–1^ (416 and 424 cm^–1^ in the theoretical spectrum) is due to pyrylium core displacements with some selenophene ring displacements and some out of plane movement. There are a number of similar selenophene ring vibrations in the 500–600 cm^–1^ region and the 960–975 cm^–1^ region, which give somewhat different enhancement profiles with different excitation frequencies.

### Surface enhanced resonance Raman scattering

**Fig. 5:**
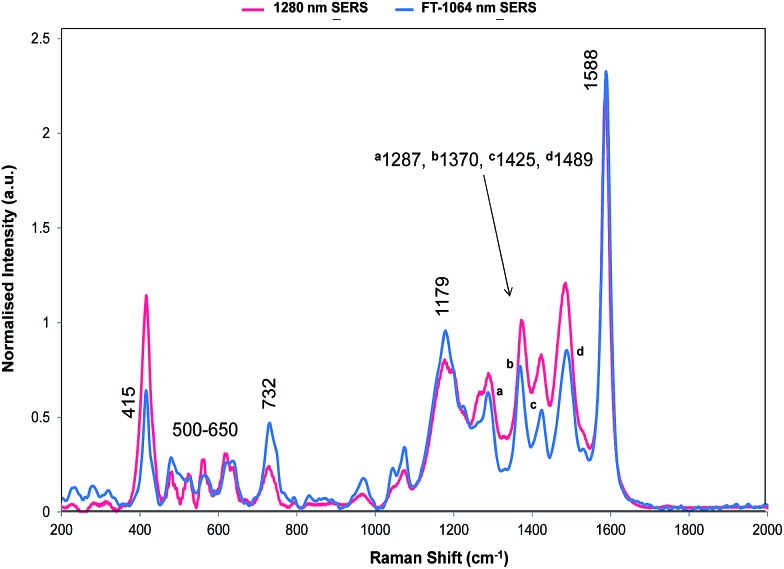
SERS of dye **1** obtained with 1064 and 1280 nm excitation. The 1064 nm Raman bands which are discussed throughout are labelled accordingly. In addition, this data has been normalised to enable the comparison between the Fourier transform 1064 nm instrument and dispersive 1280 nm instrument.

Two quite different types of spectra are obtained. The spectra excited at 785 and 633 nm are very similar to the resonance spectra (Fig. 4) but those at 1064 and 1280 nm are different and contain a number of new intense bands (Fig. 5). Spectra obtained with 785 and 633 nm excitation involve an enhancement both by molecular resonance and by surface enhancement giving surface enhanced resonance Raman scattering whereas scattering obtained using 1064 and 1280 nm excitation are enhanced mainly by surface enhancement (SERS). Excitation with 785 nm radiation provides enhancement, which is closest to the absorbance maximum at 826 nm but excitation with 633 nm excitation provides enhancement, which is closest to the plasmon maximum. In addition, in B type resonance enhancement, the transition between the 0–1 vibronic band contributes enhancement as well as the 0–0 vibronic band so if the tentative assignment of the band in the electronic spectrum at 725 nm to the first vibronic band is correct, the 633 nm Raman spectrum may also gain significant resonance enhancement from this transition. A plot of frequency against intensity expressed as a ratio of sample signal to cyclohexane signal intensity is given as an inset in Fig. 4. Since the spectra are taken from suspensions of HGNs, there may be some self-absorption of the scattered radiation and the use of different instruments with different depth of focus makes this difficult to correct, so the magnitude of the relative enhancements must be treated cautiously. However the importance of the molecular resonance contribution is clearly illustrated by the 10-fold increase in enhancement with 785 nm excitation.

The wavelength of the plasmon resonance maximum does not change significantly on aggregation (Fig. S2b†), but the SERRS enhancement is greatly increased suggesting that the SERRS intensities arise mainly from a small number of dimers or aggregates, which will have high field intensities at the interstices and different lower frequency plasmon maxima. Given the additional resonance contribution to the enhancement, scattering at 633 nm appears quite low. However, self-absorption of the scattered radiation is at a maximum at 690 nm and the contribution of scattering and absorption to the extinction is wavelength dependent with the scattering component increasing towards the infrared.

There are some differences in the SERRS spectra taken with 633 nm and 785 nm excitation compared to the resonance spectra. For example, the broad band at about 1500 cm^–1^ in resonance is more structured in SERS, the structure round the 1180 cm^–1^ band is different particularly with 633 nm excitation and a number of low frequency modes are more intense in SERRS. There are also differences between the two SERRS spectra, for example in the 1500 cm^–1^ region, in the bands about 1480 cm^–1^ and in the relative intensities of the modes between 415 and 428 cm^–1^. However, the similarity between resonance and SERRS spectra is remarkable. The concentration of dye used to obtain all SERS spectra is set to give about monolayer coverage of the HGN surface and is orders of magnitude below the resonance detection limit so the spectra are due to SERRS and not resonance from dye in solution.

The intense band at 1588 cm^–1^ shows major displacements in the thiopyrylium rings and along the long axis of the core pyrylium system (Fig. 3) giving maximum polarisation in this direction along the easily polarisable π electron cloud. Since surface enhancement arises from a change in polarisation perpendicular to the surface plane, for this band to be strong in SERRS the long axis of the pyrylium core should be at an angle close to perpendicular to the surface. The changes in the spectra at 1500 cm^–1^, at 1180 cm^–1^ and at 1425 cm^–1^ suggest that the surface may have differently oriented selenophene rings to the crystal but the SERS discussed below is more informative on this point.

The relative intensities of bands recorded with 1064 and 1280 nm excitation depend mainly on SERS selection rules with bands that have significant polarisability perpendicular to the surface plane being intense. However, resonance Raman theory predicts a drop off in intensity away from the resonance maximum which follows a Lorentzian curve. Therefore some resonant assistance to the enhancement may still occur in the spectrum obtained with 1064 nm excitation and this may account for the differences between the two spectra. New bands appear in both and the low frequency region contains fewer more intense bands than in SERRS or resonance spectra. In the high frequency region strong bands appear at 1370 and 1287 cm^–1^ and there is a significant rise in the high energy shoulder associated with the 1179 cm^–1^ band system in the 1280 nm spectrum.

There is some variation in the peak positions between the two spectra – many within the experimental error – and frequencies used here are for the 1064 nm SERS spectrum. However, the band at 1588 cm^–1^ is seven wavenumbers lower in SERS than in resonance. This is larger than would be expected due to experimental error particularly after calibration with cyclohexane so there is a shift in energy, which reflects the effect of adsorption of the ligand to the surface.

**Fig. 6:**
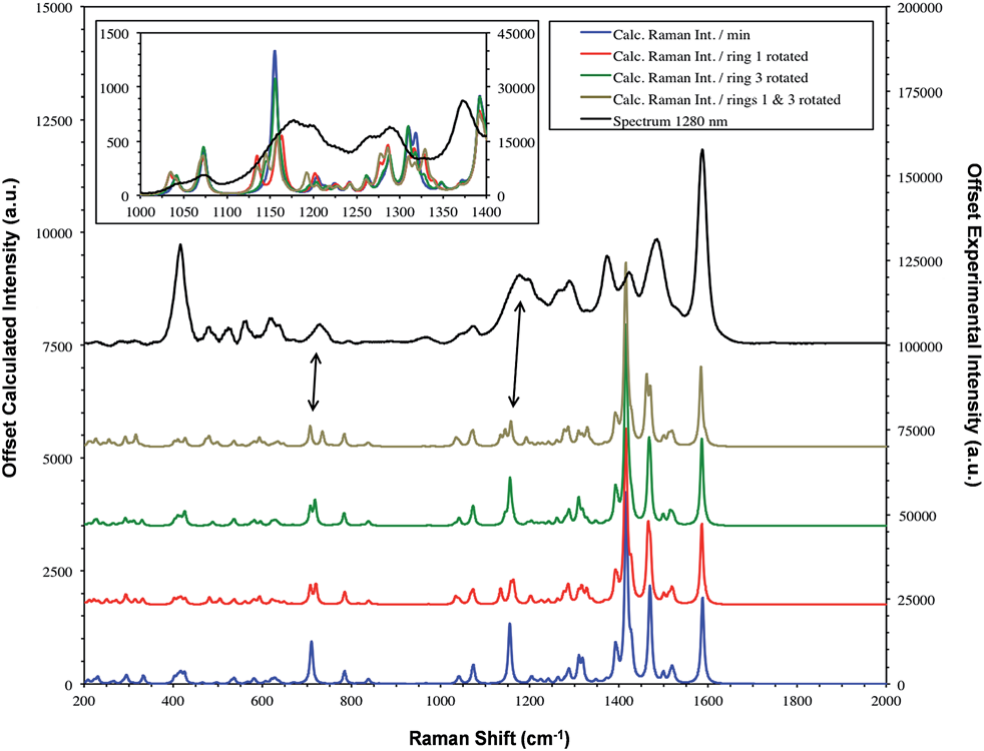
SERS spectrum of dye **1** taken with 1280 nm excitation and compared to the theoretical calculation with either the Se1 or Se3 ring or both rotated. The double headed arrows highlight the significant bands at 1179/1177 and 732/728 cm^–1^. Fig. 7 provides the extreme vibrational displacements for these modes. More extended spectra are presented in Fig. S3.†

The low barrier to rotation of the selenophene rings makes ring reorientation on adsorption to the surface very likely. Calculations of the spectral changes caused by orientation of rings one and three are shown in Fig. 6. The different selection rules for Raman scattering and SERS^32^,^33^ mean that intensities would not be expected to fit well but frequency variations are sufficiently small to use the shifts together with a study of the calculated displacements to assign the main SERS active bands.

The bands at 1370 and 1287 cm^–1^ are strong only in SERS spectra. The 1370 cm^–1^ band is assigned to the mode calculated at 1362 cm^–1^. This band has displacements on the pyrylium backbone, which extend across the bridges to the double bond opposite the selenium atoms in the selenophene rings. There are no infrared active peaks close to this frequency that could have given rise to infrared breakthrough. A number of modes between 1200 and 1300 cm^–1^ have displacements along the pyrylium backbone extending onto the four selenophene rings. The 1287 cm^–1^ mode is assigned to one of these but the modes are similar and it is likely that the surface will affect this to produce a new mode with similar characteristics in any case. The main feature of these modes, which contrasts with the previous modes discussed as intense, is the significant polarisability change which will be induced along the extension into the selenium rings. The direction of the displacement depends on the ring but is at an angle between 85° and 120° to the pyrylium core axis. To provide a reasonable polarisability perpendicular to the surface, this suggests the axis of the pyrylium core may not be exactly perpendicular to the surface.

**Fig. 7:**
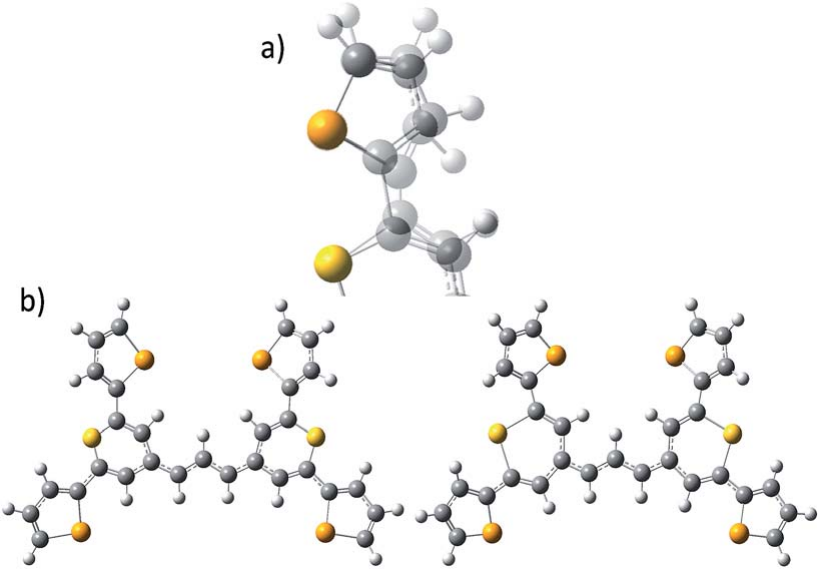
Extreme positions of the vibrational displacements for the vibration at 1179/1177 cm^–1^ (a) with the positions superimposed and for the vibration at 732/728 cm^–1^ (b) with each extreme shown separately.

The structure on the band systems at about 1179/1177 cm^–1^ and at 732/728 cm^–1^ in the 1064 and 1280 nm spectra respectively, provide useful information about the selenophene-ring orientations since the calculations give different results depending on which ring is rotated and whether one or two rings are rotated (for the extreme vibrational displacements of these modes see Fig. 7). For example, with no rings rotated one band is predicted but there is further structure with one or two rings rotated. The band at 732/728 cm^–1^ is essentially the symmetric stretch of the S-containing rings with some Se–C bond movement. The effect of rotating the rings away from the more symmetrical crystal arrangement creates two closely spaced peaks when one ring is rotated and two more widely separated peaks when two rings are rotated. This latter arrangement fits better with the experimental spectrum. The calculated spectra around 1155 cm^–1^ can be assigned to the 1179/1177 cm^–1^ band system. Although less definitive, the best fit is with two rings rotated which gives three bands over an energy range similar to the two peaks and shoulder in the experimental spectrum. These calculations also help to explain the breadth of the peaks in the resonance spectra discussed earlier.

In the 500 to 650 cm^–1^ region there are a large number of modes but many divide into two classes, some have selenophene ring displacements with some core pyrylium displacements and some are mainly pyrylium ring displacements. Both types usually have out of plane displacements. The SERS spectra contain fewer more intense peaks than the resonance spectrum. This may be due to the SERS enhancement favouring modes with significant selenophene ring and core displacements. The strong band at 415 cm^–1^ in the experimental spectrum fits better with the band predicted at 424 cm^–1^ which has pyrylium displacements and selenophene ring displacements between the selenium and the opposite C=C bond as well as some out of plane movement.

### Orientation analysis by SFG-VS

The orientation and anchoring motifs of dye **1** on a gold surface can be directly determined by SFG-VS of the dye at the gold/air interface. As the typical HGN particle size is slightly under 70 nm, one can safely assume that the surface curvature is minimal in the scale of the molecular length (∼1.5 nm). Therefore, the use of a planar gold interface can be a reliable model for the purposes of analysing the interactions between dye and the gold surface that determine the interfacial tethering and the orientation of the adsorbed dye. Studies have shown that it's not until the surface is curved on a size scale comparable to the adsorbate chain length that the molecular conformation and lateral surface interactions begin to noticeably differ from those of larger particles and extended surfaces.^34^–^37^

SFG-VS is a surface-selective vibrationally enhanced three-wave mixing process. In brief, generation of light at the sum of the infrared (IR) and visible (VIS) incoming beam frequencies is electric-dipole forbidden in centro-symmetric media but allowed at the interface where the inversion symmetry is inherently broken.^38^–^40^ SFG-VS is a coherent combination of an infrared and a Raman transitions and the SFG selection rules require for a vibrational mode to be infrared as well as Raman active.

**Fig. 8:**
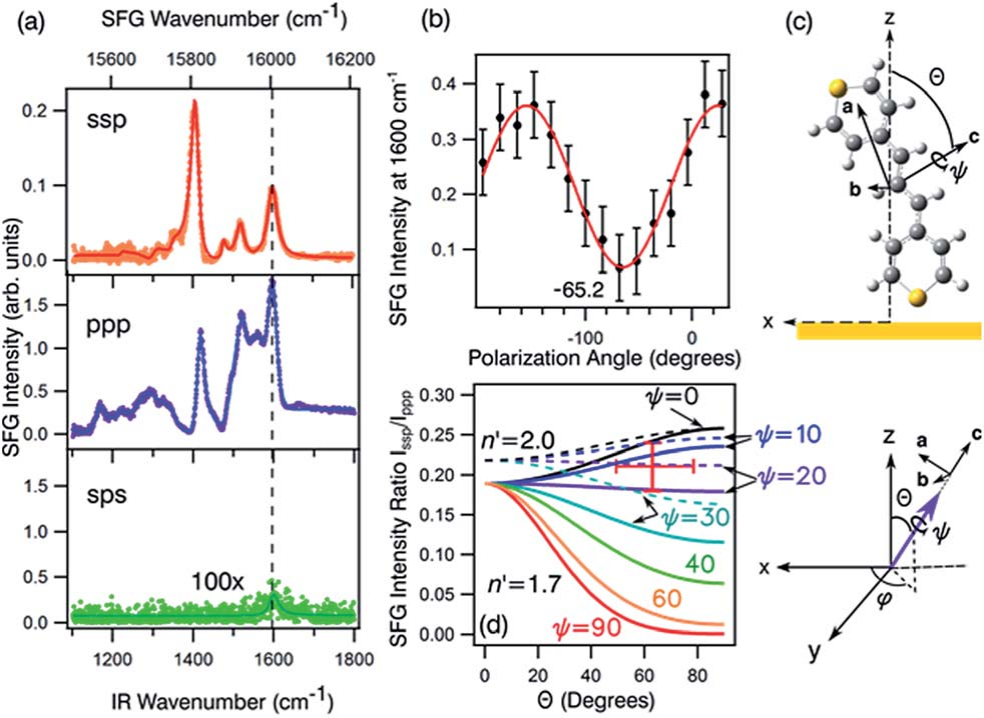
(a) The ssp, ppp, and sps SFG spectra of dye **1** on a polycrystalline gold film. (b) PNA results for the 1600 cm^–1^ resonance showing a minimum at a polarisation angle of –65.2°. (c) The molecular frame coordinate system used for orientation analysis of the pyrylium backbone and the relationship between the macroscopic laboratory frame (upper) and microscopic coordinate system (lower). The net transition dipole of the molecular fragment is aligned with the *c*-axis which is also the symmetry axis. *θ* describes the angular tilt of the transition dipole from the surface normal and *ψ* is the twist angle of the pyrylium backbone about the symmetry axis. (d) Simulation of the ssp to ppp SFG intensity ratio for the 1600 cm^–1^ symmetric stretch. The solid lines are calculated using a refractive index for the interface of *n*′ = 1.7 and the dashed lines of *n*′ = 2.^51^ The red cross-hair indicate the experimentally determined ratio (vertical) while the horizontal mark represents most probable tilt angles as described in the text.

Orientation from various functional groups at the interface can be inferred from a series of polarisation combinations, which provide access to the different elements of the second-order nonlinear susceptibility *χ*^(2)^ tensor.^41^,^42^ The macroscopic susceptibility tensor elements can be then described by a combination of the three optical fields (SFG, VIS, and IR). Linearly polarised light at the surface is described as s-polarised or p-polarised depending whether the electric field vector is perpendicular or parallel to the interface, respectively. With that convention, s-polarised light contains only a *y*-component while p-polarised contains *x*- and *z*-components of the optical field in the laboratory frame (Fig. 8). For an achiral molecular surface, all non-zero elements of the second-order susceptibility can be accessed by three polarisation configurations: ssp, ppp, and sps, where the indexing order corresponds to the SFG, VIS, and IR beam polarisation, respectively. In general, the ssp and sps polarisation combinations can be utilised to probe vibrational transitions oriented mainly perpendicular and parallel to the surface through the χ(2)yyz and χ(2)yzy elements, respectively, while the ppp polarisation combination is a coherent superposition involving four tensor elements (χ(2)xxz, χ(2)xzx, χ(2)zxx and χ(2)zzz).^41^,^43^

Fig. 8a shows the SFG spectra in ssp, ppp, and sps polarisation collected for the as-spun dye **1** on a gold substrate in the 1100–1800 cm^–1^ region (access to lower wavenumbers was limited by the mid-IR laser power). A VIS wavelength of ∼694 nm was chosen in order to generate SFG signals between 615 and 645 nm (shown as wavenumbers in the upper axis in Fig. 8a). When the SFG frequency matches an electronic resonance of the adsorbed species, doubly-resonant enhancement may be observed, analogous to resonance Raman.^44^ The SFG wavelengths here fall in a zone of minimal absorption, purposely chosen to simplify the orientational analysis.^43^ The ssp spectrum obtained shows remarkable similarity with the calculated Raman spectra in Fig. 3 and 6, with main contributions at 1599, 1528, 1476 and 1406 cm^–1^ corresponding to the modes described in Table S1 in ESI.† It is interesting to note that the peaks below 1400 cm^–1^, which resemble the Raman peaks in this region are predominantly shown in the ppp polarisation, suggesting a different symmetry for the vibrational transitions compared to those prominent in the ssp polarisation combination. A peak at 1560 cm^–1^ is clearly observed in the ppp signal while being barely noticeable in the ssp spectrum. The ppp response shows a larger non-resonant contribution which interferes with the resonant features giving rise to complex line shapes as commonly observed in SFG-VS of metal surfaces.^38^,^45^–^48^ A single feature at 3098 cm^–1^ was observed in the CH stretching region as shown in Fig. S4, ESI.† The orientation at a surface is deduced from the ratio of the SFG intensity of resonant components at various polarisation configurations. The spectra were fitted to a sum of complex Lorentzians to quantify the individual vibrational contributions to each spectrum according to:^45^,^47^,^49^ 
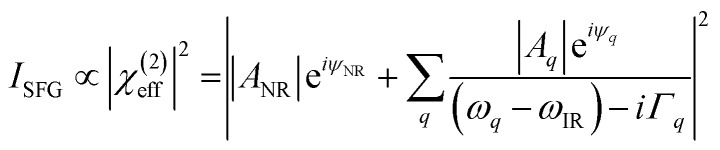
where *χ*(2)eff is the effective susceptibility for a particular polarisation combination *A*_*q*_ and is the amplitude of resonance frequency *ω*_*q*_ with a full width at half-maximum (FWHM) of 2*Γ*_*q*_ and phase angle *ψ*_*q*_ (this phase angle is usually either 0 or π for non-metallic substrates). The sub-index NR stands for the non-resonant background. Compared to dielectric substrates, where the non-resonant signal is negligible, the additional complications induced by the large contribution of the background signal in metals (and some semiconductors) has restricted the wide-spread use of SFG quantitative analysis on these surfaces.^41^,^43^–^45^ To correctly compare the intensities of the resonances, the individual SFG intensities are calculated according to *I*_*q*_ = (*A*_*q*_/*Γ*_*q*_)^2^. The ratios of these values in different polarisation combinations can be plotted against curves like the ones shown in Fig. 8d to determine the average orientation at the surface.

Emphasis is now placed only on the 1599 cm^–1^ mode, attributed to the C=C symmetric stretches in the thiopyrylium core. For this mode we assume *C*_2v_ symmetry where the symmetry axis is aligned with the vibrational transition dipole (the *c*-axis in the molecular frame shown in Fig. 8c). Such arrangement allows us to directly determine the orientation of the thiopyrylium core from our SFG analysis. This approach is similar to recent SFG studies of the C=C stretching mode of polythiophene films for determining interfacial ring orientation.^50^–^52^ The molecular frame used for the pyrylium core follows standard convention with the molecular *c*-axis lying along the direction of the net transition dipole (which falls in the average plane of the core), the *a*-axis also is in the plane of the pyrylium backbone and is oriented along the two aromatic units, and the *b*-axis is orthogonal to *a* and *c*. It is not expected that the selenophene substituents will induce a meaningful distortion to this symmetry and are left out of the SFG orientation calculation for the 1600 cm^–1^ mode for simplicity. The amplitude of this mode can be related to the macroscopic susceptibility terms and their respective relation to the microscopic hyperpolarisability elements can be used to determine the orientation of the dye backbone. This relationship is given by:^41^,^43^ 
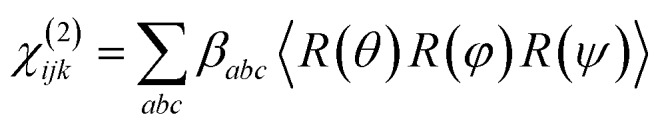
 *β*_*abc*_ are the hyperpolarisability elements in the molecular frame and are the hyperpolarisability elements in the molecular frame and 〈〉 indicating the average of the molecular orientation over the distribution. are the hyperpolarisability elements in the molecular frame and 〈〉 indicating the average of the molecular orientation over the distribution. indicating the average of the molecular orientation over the distribution. *R*(*θ*), *R*(*φ*), and *R*(*ψ*) represent the Euler rotation matrices transforming the molecular coordinate system into the laboratory fixed frame. These angles are indicated in Fig. 8c. The exact form of the effective susceptibility elements for the *C*_2v_ point group can be found elsewhere^41^ and consist of orientational averages of hyperpolarisability tensor components multiplied by the local field weighing factors. For a symmetric stretch in a *C*_2v_ point group, the non-zero hyperpolarisability components are *β*_*aac*_, *β*_*bbc*_, and *β*_*ccc*_. Using DFT calculations for the chalcogenopyrylium core, we obtain *β*_*aac*_ ≫ *β*_*ccc*_ > *β*_*bbc*_ (*ca.* 10 : 1 : 0.1). This result is analogous to the calculations by Anglin *et al.* for a methylthiophene dimer where the large change in polarisability along the *a*-axis was explained as a likely consequence of the extended conjugation in this dimension.^50^

With these assumptions, the predicted variation for the intensity ratio *I*_ssp_/*I*_ppp_ with respect to the azimuthal angle *θ* and twist angle *ψ* about the symmetry axis is illustrated in Fig. 8d. For these calculations, a rotationally isotropic surface is assumed and curves are averaged over *φ*. The optical constants used for the local field corrections are provided in the ESI.† The intensity ratio determined for the 1599 cm^–1^ mode from the Lorentzian fits is *I*_ssp_/*I*_ppp_ = 0.18 ± 0.05. Complementary to this measurement, we also applied the polarisation null angle method (PNA), which is given by the relationship: tan *Ω*_null_ = *χ*_ppp_/*χ*_ssp_ = 
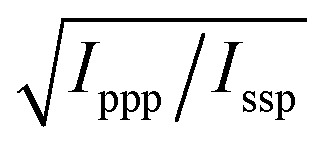
. The PNA method can be more accurate than the fitting approach to determine the intensity ratio.^53^–^58^ Fig. 8b shows the SFG intensity for the 1599 cm^–1^ mode. Because of the non-resonant background, the spectrum obtained at each polarisation angle is fitted to a sum of Lorentzians as described above and the intensity determined for this mode is plotted as a function of the polarisation angle in Fig. 8b. The data shows that this intensity can be almost fully extinguished, when the polariser analyser is set to –65.2 ± 1.7°, this translates into *I*_ssp_/*I*_ppp_ = 0.21 ± 0.03, which is in agreement with the results obtained from the fitting. The spectral measurements in Fig. 8a and the PNA experiments in Fig. 8b were carried out on different days, showing great reproducibility in the experiments.

Based on these results, we can confidently rule out the possibility that the pyrylium backbone is strictly perpendicular to the surface (*ψ* = 0) since our calculated ratio is statistically lower than the predicted values for such orientation. We can also exclude the possibility of the molecule lying flat on the surface (*θ* = 90° and *ψ* = 90°) since our intensity ratio is remarkably higher. Possible orientations that would generate ssp and ppp signals with the measured ratio can have a number of combinations of *θ* and *ψ*. From these, we can safely rule out *ca.* 80° < *θ* < 50° on energetic arguments, since it would imply that the H atoms from one selenophene substituent point towards the surface while having both the Se and S atoms away from the interface within the allowed twist angles. This logic reduced the possible orientations of dye **1** at the surface to *θ* ∼ 50–80° and corresponding *ψ* falling between 0° and 30°, which relates to the molecule standing up on one end with the S atom pointing towards the interface (this determined by *θ*) and making an angle of 90° – *ψ* with respect to the plane of the surface. When the aromatic ring in proximity to the interface is aligned vertically to give the closest approach of the S atom to the surface (as shown pictorially in Fig. 8c), the determined tilt angles of the thiopyrylium backbone fall in the range of 64–85° from the surface plane (*ψ* = 5–26°). Most of the uncertainty in this calculation originates from our estimation of the index of refraction of the interfacial layer as illustrated in Fig. 8d. The sps signal is very weak and was not used for orientational analysis due to the low signal to noise ratio, however, the intensity is non-zero demonstrating a parallel component of the transition dipole.

### Orientation and bonding on the surface

**Fig. 9:**
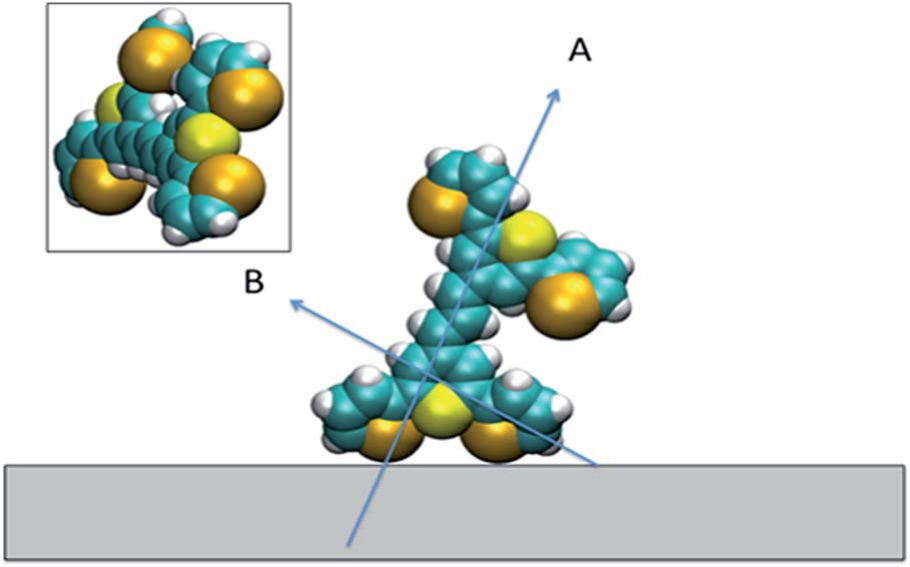
Space filled model of the dye on the surface showing the two selenophene rings rotated and the position of the thiopyrylium S atom. Arrow A indicates the long axis of the thiopyrylium core and arrow B the direction of the bridge from one selenophene ring to the core. The inset shows the molecule rotated to show the relative positions of the Se and S atoms facing the surface.

For all the SE(R)RS studies, the HGNs had a monolayer coverage of the analyte. Therefore the dye was not sparsely populated on the HGN surface. This packing stabilisation forced by reporter–reporter interactions, increases our confidence in that similar orientations where obtained in both the SFG-VS and SE(R)RS experiments enabling us to conduct the comparison between the two techniques. We must be mindful however of the differences in the environments such as curvature of the HGNs *versus* the planar surface of the dried film and also the obvious difference between the air/metal and water/metal conditions used. Therefore, to further help in our understanding of how dye **1** orientates on the HGN surface and to build on the information obtained from these two techniques, a space filled model of the molecule with rings Se1 and Se3 rotated was calculated and the prediction given in Fig. 9. Arrow A shows the angle of the long axis of the thiopyrylium core to the surface as indicated by SERRS. This is further confirmed by SFG-VS using a dried film. SFG-VS defines the angle of the major axis as between 64° and 85° which is compatible with the suspension SERRS/SERS data. In this orientation the main displacement direction for the SERS bands discussed between 1370 and 732 cm^–1^ is at an angle to the surface (arrow B) as are the ring displacements found in low frequency modes such as that giving rise to the band at 415 cm^–1^. Thus SERRS with a strong dependence on molecular resonance will give intense bands due to significant polarisability perpendicular to the surface along the major axis from modes which produce strong molecular resonance enhancement and SERS will give intense bands from modes with displacements along the same axis and in addition from the displacements along the direction of arrow B and on the selenium rings.

The low barrier to rotation means that the precise angle of the rings to the surface will be determined by surface bonding and packing forces. The two selenium atoms and the sulphur atom would be expected to bond to the gold surface since each is a soft base but the angles of the rings cannot be set so that the sulphur and selenium atoms can all point directly at the surface to form simple linear bonds. Furthermore, the thiopyrylium sulphur atom is somewhat protected from the surface by the selenophene rings, see inset in Fig. 9. However, the forces holding gold atoms in a plane on the surface are weak and it may be that gold atoms are displaced to bond between the selenium atoms. Thus, the interaction is likely to be complex involving gold displacements and metal to ligand π as well as σ bonding. This model fits with the SERS data. There are differences in frequency for some modes, particularly the 1588 cm^–1^ mode, between SERS and resonance as a result of surface bonding. Some modes are broadened as predicted by the calculations following reorientation of the selenophene rings. These will also be affected by packing and surface interactions. The position of the two selenophene rings away from the surface is difficult to define. However, the backbone position suggests that, since enough dye was added to reach about monolayer coverage, efficient packing of layers of dye **1** is likely and the low barrier to rotation of the rings suggests that they will accommodate the most effective packing arrangement.

## Conclusions

The nature of the bonding of a label to a metal surface in aqueous suspension at monolayer coverage is difficult to probe but some techniques are able to give partial answers, which are much more robust when different techniques are combined. SFG-VS provides good evidence of the orientation of the label at the air-gold interface. The SERRS data at the water–gold interface describes a similar orientation. The SERS then adds detail on the ring orientation and nature of the surface interaction, giving a model which can be used to plan future synthesis strategies to increase the range and effectiveness of this new set of labels.

In addition, these spectra clearly show the different enhancement mechanisms involved in SERRS and SERS. In SERRS, the very polarisable π system involved in the electronic transition couples well with the plasmon to give strong SERRS from vibrations which also benefit from strong molecular resonance enhancement. However, well away from excitation frequencies, which give SERRS, where SERS intensities are dominated by the induced polarisability perpendicular to the surface, new bands appear which reveal more structural information.

## Supplementary Material

Supplementary information
