# Supplementary material for: Elucidation of the bonding of a near infrared dye to hollow gold nanospheres – a chalcogen tripod
Source: Chem Sci. 2016 Apr 21;7(8):5160–70. doi: 10.1039/c6sc00068a (PMC6020253; doi:10.1039/c6sc00068a)
Supplement: Supplementary file 1 [file SC-007-C6SC00068A-s001.pdf]

Electronic Supplementary Material (ESI) for Chemical Science.
This journal is © The Royal Society of Chemistry 2016

Elucidation of the Bonding of a Near Infrared Dye to Hollow Gold Nanospheres – A Chalcogen Tripod

Hayleigh Kearns,[a] Sanghamitra Sengupta,[b] Ivan Ramos Sasselli,[a] Leander Bromley, III,[b] Karen Faulds,[a] Tell Tuttle,[a] Matthew A. Bedics,[b] Michael R. Detty,[b] Luis Velarde,[b]* Duncan Graham[a]* and W. Ewen Smith[a]*

Table S1. Assignment of main bands (cm^{-1}) in the resonance and SERS spectra using the corrected calculation.

Theor. Raman	Expt Raman 785 nm	SERS FT-1064 nm	SERS 1280 nm	Assignments for Dye 1
416; 424 (doublet)	415 (br)<br><br>428; 410 (sh)- 633 nm	415	416	416; Out of plane rocking movement on bridge with some selenophene 3 & 4 movement<br>424: Stretch of S-containing rings (C-S), (C=C), (C-H) with rocking movement on bridge (C=C), (C-H)
464; 486 (w)	472	479	478	Stretch of S-containing rings (C-S), (C=C), (C-H) and all four selenophene rings (C-Se), (C=C), (C-H) (486– selenophene rings 1 & 2)
497; 536 (w)	500 (633nm-w)	527	523	Elongation of all four selenophene rings (C-Se), (C=C), (C-H) with bending of S-containing rings (C-S), (C=C), (C-H) – out of plane<br>(497 – selenophene rings 3 & 4)
581	568	565	559	Rocking movement on bridge (C=C), (C-H) - out of plane
607 (w)	619	620	616	Extended vibration in core (C=C), (C-H) with elongation in S-containing rings (C-S), (C=C), (C-H)
628	642	636	638 (sh)	Rocking movement on selenophene rings 1 & 3 (C-Se), (C=C), (C-H)
710	735	732	728	S-containing ring breathing mode (C-S), (C=C), (C-H); extended to selenophene rings (C-Se), (C=C), (C-H)
969 (w)	965(633nm-w)	971	965	selenophene rings (C=C), (C-H)
1041	1044	1044	1044	selenophene rings 3 & 4 (C-Se), (C=C), (C-H)
1073	1078	1073	1071	(C-H) bending in selenophene rings with rocking movement throughout bridge, core (C=C), (C-H) and Se rings (C=C)
1155; 1204	1180 (br)	1179 (br) 1223 (sh)	1177 (br)	Core (C=C), (C-H) with extension through bridge (C=C) into selenophene rings (C-

			1200 (sh)	Se), (C=C), (C-H)
1263; 1288	1246 1297	1287	1265(sh) 1288	Bridge (C=C) to selenophene rings 2 & 4 with less movement in selenophene 1 (C-Se), (C=C), (C-H)
1362 (w)	1360 (w)	1370	1372	Core (C=C), (C-H) extending to (C=C) bridge to selenophene rings 3 & 4 (C-Se), (C-H)
1416	1425; 1417 (sh)	1425	1423	selenophenee ring (C-Se), (C=C), (C-H) to S-containing ring movement (C-S), (C=C), (C-H) (mainly selenophene 1 & 2) and core (C=C)
1470;	1487	1489	1485	Core (C=C), (C-H) and S-containing rings (C-S), (C=C), (C-H) with extension to selenophene rings (C-Se), (C=C), (C-H)
1500; 1518	1507; 1535	1530 (sh)	1526 (w)	Core (C=C), (C-H) and selenophene rings (C-Se), (C=C), (C-H); (1518 – selenophene rings 3 & 4; 1500 – selenophene rings 1 & 2)
1588	1595	1588	1587	Core (C=C), (C-H) and S-containing rings (C-S), (C=C), (C-H)

sh = shoulder; br = broad; w=weak

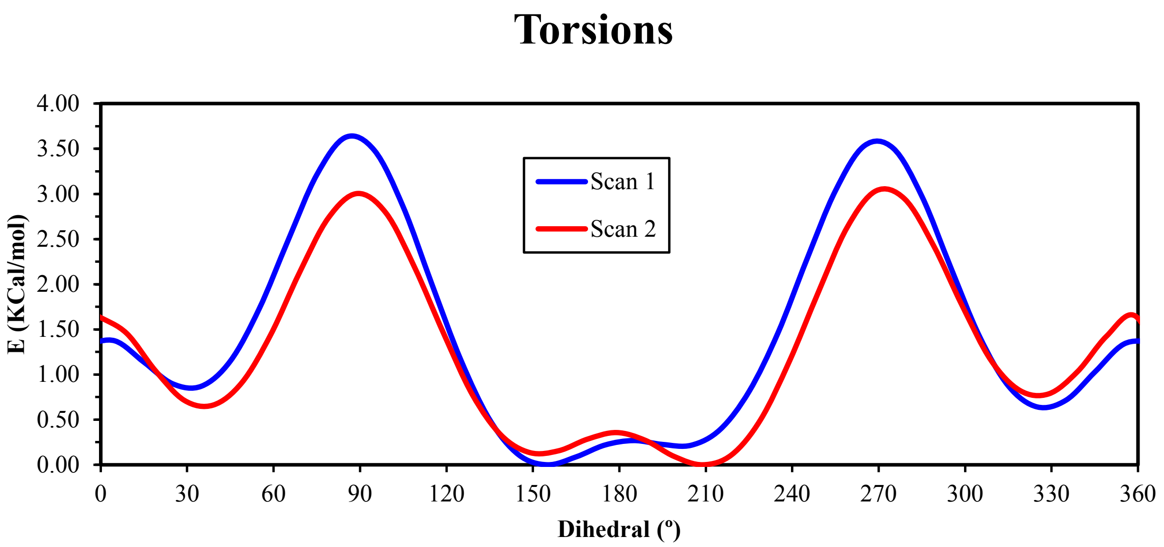

Figure S1. Torsion angles demonstrating the barriers to rotation of the rings is ~4 kcal. Scan 1 = Se - C - C – S; scan 2 = Se - C - C – S.

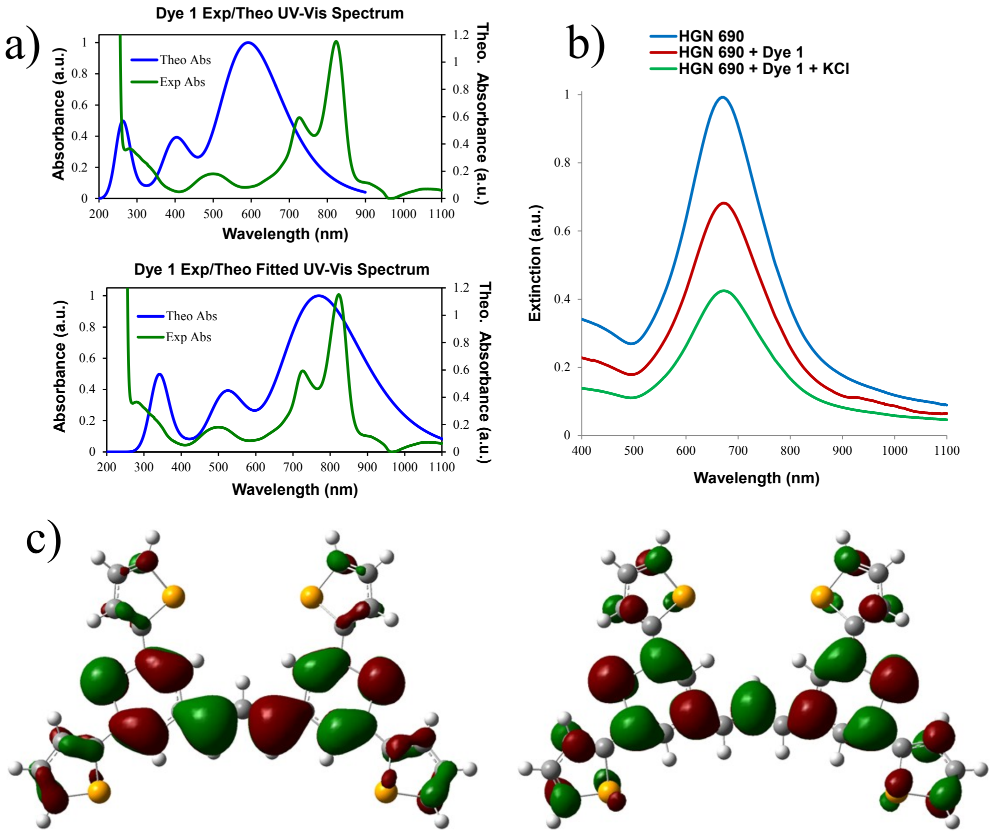

Figure S2. a) Calculated (blue) and experimental (green) spectra for dye **1** b) extinction spectrum for the HGN alone, with dye **1** added and with the labelled HGN aggregated c) electron density of the π (left) and π* (right) orbitals which give rise to the 826 nm band in a) and b).

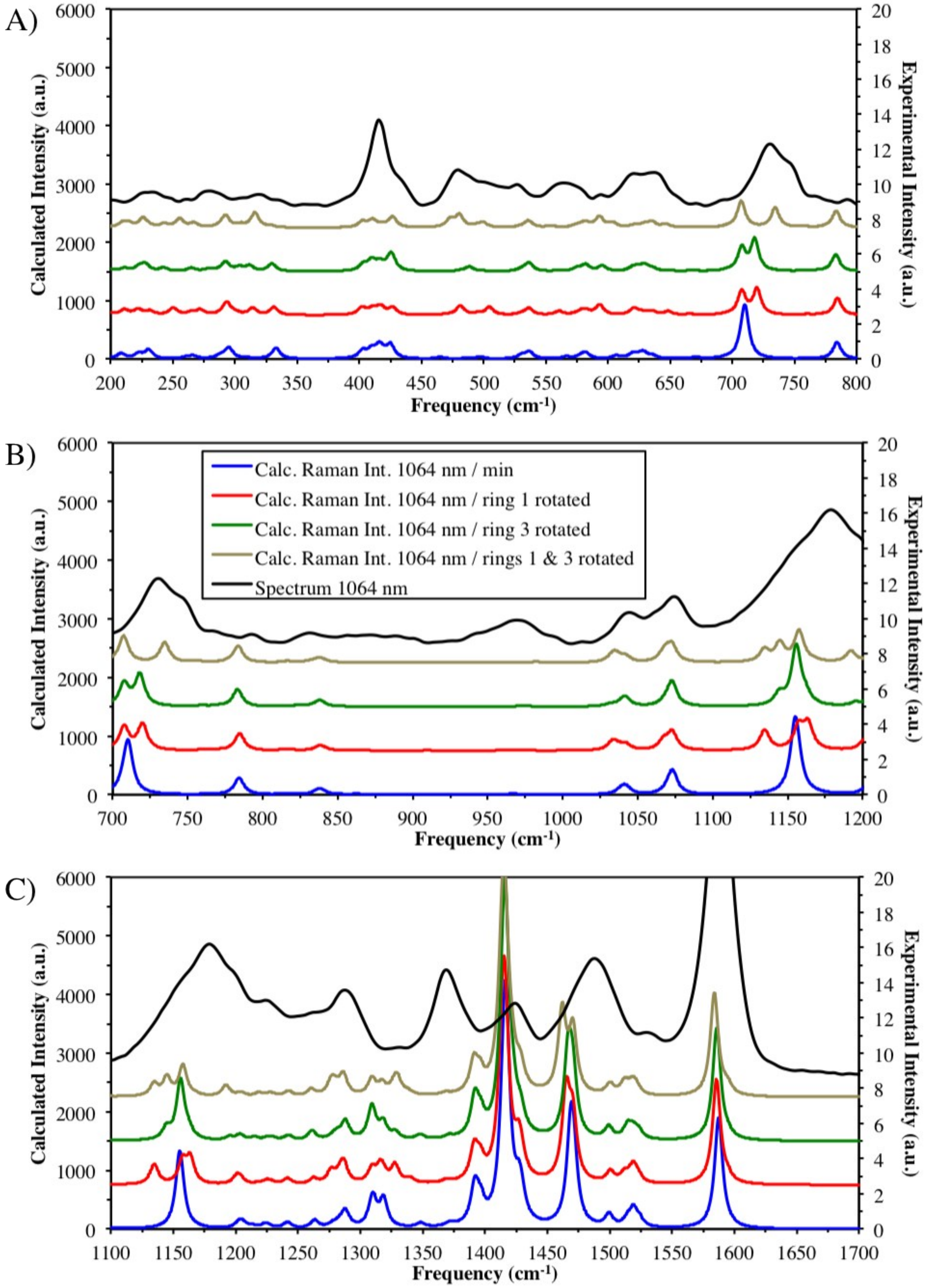

Figure S3. Expanded theoretical and experimental 1064-nm Raman spectra.

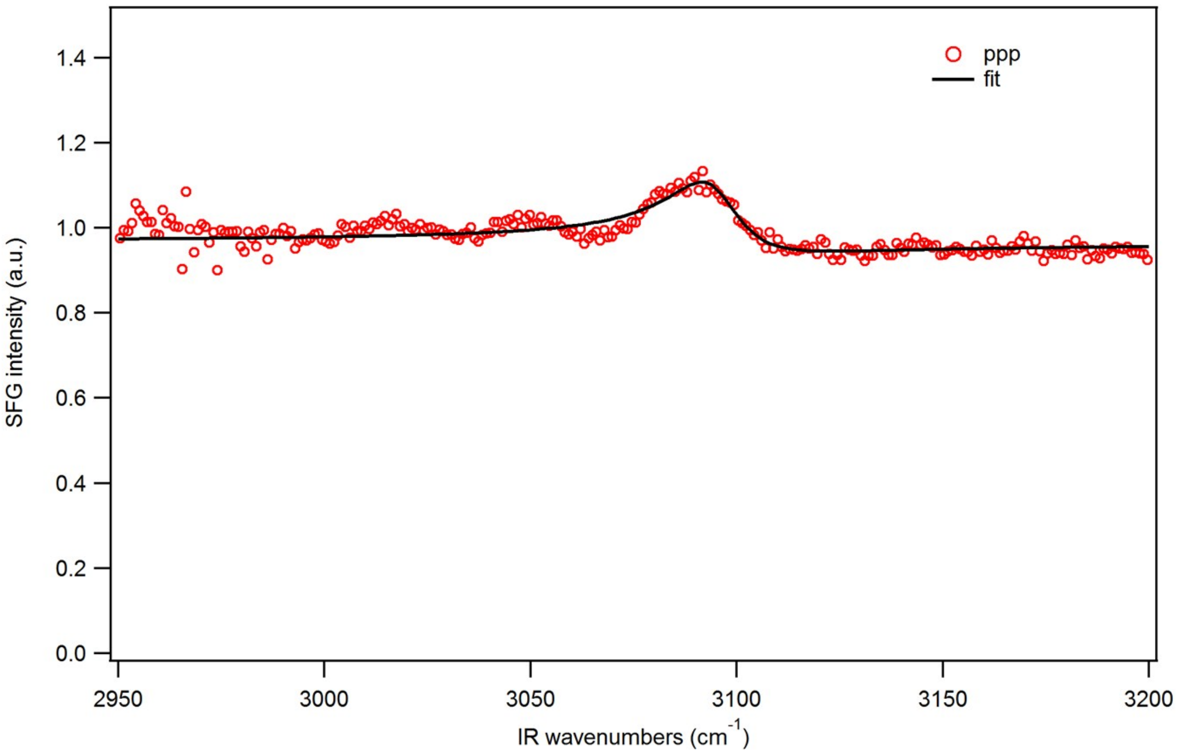

Figure S4. A single weak spectral feature in the ppp SFG spectrum is observed at 3098 cm^{-1} and attributed to the various C-H stretches of dye-1.

Table S2. Refractive index of gold and the interface

refractive index	Gold[1]	n' (interface)[2,3]
**IR at 1600 cm^{-1}**	4.599 + 42.577*i*	1.7-2
**visible at 694 nm**	0.135 + 4.070 *i*	1.7-2
**SFG at 625 nm**	0.167 + 3.475 *i*	1.7-2

References

1. R. L. Olmon, B. Slovick, T. W. Johnson, D. Shelton, S.-H. Oh, G. D. Boreman and M. B. Raschke, *Phys. Rev. B*, 2012, **86**, 235147
2. P. Dhar, P. P. Khlyabich, B. Burkhart, S. T. Roberts, S. Malyk, B. C. Thompson and A. V. Benderskii, *J. Phys. Chem. C*, 2013, **117**, 15213–15220
3. H. Zhang , C. Romero and S. Baldelli, *J. Phys. Chem. B*, 2005,**109**, 15520–15530
